# LncRNA HOTTIP Knockdown Attenuates Acute Myocardial Infarction via Regulating miR-92a-2/c-Met Axis

**DOI:** 10.1007/s12012-021-09717-3

**Published:** 2022-01-19

**Authors:** Beilei Wang, Likun Ma, Junyi Wang

**Affiliations:** 1 Department of Cardiology, the First Affiliated Hospital of USTC, University of Science and Technology of China, Division of Life Sciences and Medicine, No.17 Lujiang Road, Hefei City, 230036 Anhui Province People’s Republic of China; 2 Institute of Ageing Research, Hangzhou Normal University School of Medicine, Hangzhou City, 311121 Zhejiang Province People’s Republic of China

**Keywords:** AMI, HOTTIP, miR-92a-2, c-Met, Apoptosis

## Abstract

Increasing investigations have focused on long non-coding RNAs (lncRNAs) in various human diseases, including acute myocardial infarction (AMI). Although lncRNA HOTTIP has been identified to play an important role in coronary artery diseases, its role and specific mechanism in AMI remain unclear. To investigate the potential role of HOTTIP in MI, HOTTIP expression in hypoxia-treated cardiomyocytes and myocardial tissues of MI mice was evaluated. The potential targets of HOTTIP and miR-92a-2 were predicted using Starbase and Targetscan. To further determine the cardio-protective effects of HOTTIP in vivo, si-HOTTIP and miR-92a-2 mimics were individually or co-injected into mice through intramyocardial injection. Moreover, their roles were further confirmed in rescue experiments. HOTTIP was significantly upregulated in ischemic myocardium of MI mice and hypoxia-induced cardiomyocytes. Moreover, HOTTIP knockdown markedly promoted cardiomyocyte growth and inhibited cardiomyocyte apoptosis in vitro. Luciferase reporter assay showed that HOTTIP could directly sponge miR-92a-2 to negatively regulate miR-92a-2 expression. In addition, c-Met was identified as a direct target of miR-92a-2, and their correlation was confirmed by luciferase reporter assay. MiR-92a-2 overexpression significantly enhanced the protective effect of HOTTIP knockdown against AMI through partially inhibiting c-Met expression. Our results demonstrated that HOTTIP downregulation attenuated AMI progression via the targeting miR-92a-2/c-Met axis and suggested that HOTTIP might be a potential therapeutic target for AMI.

**Supplementary Information:**

The online version contains supplementary material available at 10.1007/s12012-021-09717-3.

## Introduction

Cardiovascular disease has become the number one cause of death in the United States. Approximately over half of these deaths are due to acute myocardial infarction (AMI) [1, 2]. Myocardial infarction (MI) is a leading cause of heart failure and a severe threat to human lives [3, 4]. It has been reported that cardiomyocyte apoptosis is closely associated with the pathogenesis of MI [5, 6] and is one of the mechanisms underlying AMI progression. Therefore, a better understanding of cardiomyocytes apoptosis is more urgent and may contribute to identifying potential therapeutic targets for AMI.

Long non-coding RNAs (lncRNAs), a newly identified class of non-protein-coding RNAs with more than 200 nucleotides in length, have been reported to participate in multiple heart diseases [7–9]. LncRNA HOTTIP is most abundantly expressed in various human cancers and plays an important role in cancer progression. For example, HOTTIP facilitates invasion, migration, and epithelial-mesenchymal transition (EMT) of osteosarcoma cells by forming a positive feedback loop with c-Myc [10]. HOTTIP confers colorectal cancer cell metastasis and invasion by downregulating DKK1 [11]. HOTTIP promotes proliferation, invasion, and migration of papillary thyroid carcinoma cells via directly targeting miR-637 [12]. HOTTIP promotes endothelial cell proliferation and migration by activating the Wnt/β-catenin signaling pathway [13]. Recently, HOTTIP has been identified to effectively alleviate oxygen–glucose deprivation-induced neuronal injury through regulating the miR-143/hexokinase 2 pathway [14]. However, the role of HOTTIP in AMI remains unclear.

C-Met, a receptor tyrosine kinase of hepatocyte growth factor (HGF), is expressed in various cells such as epithelial cells, hematopoietic cells, and neurons [15]. Many studies have revealed that aberrant activation of the HGF/c-Met signaling pathway is closely involved in cell proliferation, metastasis, and worse prognosis of patients with different gastrointestinal cancers [16], prostate cancer [17], non-small-cell lung cancer [18], colorectal cancer [19] and gastric cancer [20]. Sato et al. revealed that c-Met was significantly upregulated in the myocardium of MI patients, suggesting that c-Met directly affects the myocardium of MI patients [21]. In addition, c-Met/HGF signaling has beneficial effects against myocardial infarction and endothelial dysfunction through its pro-angiogenic, anti-inflammatory, and anti-fibrotic functions [22]. However, the regulatory network involving c-Met in AMI has not been well studied.

This study demonstrated that HOTTIP downregulation efficiently attenuated AMI progression through inhibiting cardiomyocyte apoptosis by directly targeting the miR-92a-2/c-Met axis, suggesting that HOTTTIP might be a potential target for AMI treatment.

## Materials and Methods

### Clinical Sample Acquisition

Venous blood samples were collected from 42 MI patients (average age: 54.2 ± 4.1, male/female = 22/19) and 42 healthy volunteers (average age: 54.6 ± 3.2, male/female = 23/21) in the First Affiliated Hospital of University of Science and Technology of China. After centrifugation, serum samples were collected and used for quantitative real-time polymerase chain reaction (qRT-PCR) to detect the expression patterns of lncRNA HOTTIP.

### Mouse Model of MI

C57BL/6 mice (male, approximately 8–20 weeks) were obtained from Beijing Vital River Laboratory Animal Technology Co., Ltd. and kept under standard conditions. Mouse MI model was constructed through left anterior descending coronary artery (LAD) occlusion as previously described [23]. Mice were randomly divided into 5 groups, including sham group, MI group, si-HOTTIP group, miR-92a-2 group (miR-92a-2 mimics), and combined group (si-HOTTIP and miR-92a-2 mimics) with 7 mice in each group. 50 nM of these si-RNAs or mimics were injected into left ventricles through intramyocardial injection each day for 1 week. Mice in all groups except the sham group were subjected to MI operation. At 12 h after MI, blood samples were collected for detection of serum myocardial enzyme activities. After that, mice were euthanized, and myocardial tissues were collected for the subsequent experiments. All procedures were approved by the Ethical Committee of the First Affiliated Hospital, Division of Life Sciences and Medicine, University of Science and Technology of China.

### Cell Isolation and Culture

Mouse cardiomyocytes were isolated from 1 to 3-day-old neonatal C57BL/6 mice as previously described [24]. Briefly, the dissected hearts from mice were rinsed with HEPES-buffered saline solution, cut into pieces, and digested with 0.25% trypsin. Subsequently, cells were maintained in Dulbecco’s modified Eagle medium/F-12 (DMEM/F-12; Gibco, Carlsbad, CA, USA) containing 5% fetal bovine serum (Hyclone, Logan, UT, USA), 0.1 mM ascorbate in insulin-transferring-sodium selenite media supplement (Sigma, St. Louis, MO), 100 U/ml penicillin, 100 μg/ml streptomycin, and 0.1 mM bromodeoxyuridine (Gibco) in an atmosphere at 37 °C with 5% CO_2_.

### Hypoxia/Re-oxygenation (H/R) Injury

H/R injury in cardiomyocytes was induced as previously described [25]. In brief, cardiomyocytes were cultured with serum-free medium under normoxic conditions for 24 h. Cells were then cultured under hypoxia (5% CO_2_, 95% N_2_) for different times (12, 24, and 48 h) and subsequently under re-oxygenation condition (5% CO_2_, 95% O_2_) for another 4 h to stimulate H/R injury. In addition, control cells were cultured under normoxic conditions all the time. Finally, cells were harvested for the subsequent experiments.

### Cell Transfection

Si-HOTTIP (siRNA against HOTTIP, 5′-AGGCTGAGCTAATACAGTA-3′), si-NC, miR-92a-2 mimics, miR-NC, and si–c-Met (siRNA against c-Met, 5′-ATCTTGAGCCATTCACCGGAA-3′) were purchased from Shanghai GenePharma Co., Ltd. (Shanghai, China) and transfected into mouse cardiomyocytes using Lipofectamine 2000 (Invitrogen). To overexpress HOTTIP and c-Met, the cDNA sequence of HOTTIP and c-Met were amplified with mouse genome as the template and cloned into pcDNA3.1 Expression vector to generate pc-HOTTIP and pc-c-Met. Cells transfected with the empty vector were used as the negative control (pc-NC). Similarly, pc-HOTTIP, pc-c-Met, or pc-NC was transfected into mouse cardiomyocytes using Lipofectamine 2000.

### CCK-8 assay

Cell viability was evaluated using a Cell Counting Kit-8 (CCK-8, Dojindo Molecular Technologies, Gaithersburg, MD) as previously described [26]. Briefly, approximately 3 × 10^3^ transfected cardiomyocytes were seeded into 96-well plates and incubated with additional CCK-8 reagent at 0, 24, 48, 72, and 96 h for another 4 h. Finally, the absorbance at 450 nm was detected with a microplate reader.

### RNA Immunoprecipitation (RIP) Assay

To determine the interaction between HOTTIP and miR-92a-2, RIP assay was performed using the EZMagna RIP Kit (Millipore) following the manufacturer’s instructions. Briefly, cells lysates were incubated with magnetic beads conjugated to Ago2 antibody or negative control IgG (Abcam) overnight at 4 °C. On the next day, cell lysates were incubated with 30 μL of magnetic beads for another 2 h at 4 °C, and the immune-precipitated RNAs were purified and used for qRT-PCR analysis.

### RNA Extraction and qRT-PCR Analysis

Total RNAs were extracted from myocardial tissues of MI mice or cultured cells using TRIzol Reagent (Invitrogen, Carlsbad, CA, USA). After reversely transcribing into cDNA using the PrimeScript RT reagent Kit (TaKaRa), qRT-PCR analysis was carried out using SYBR Green PCR Kit (Thermo) on an ABI 7500 Fast Real-Time PCR system (Applied Biosystems, Carlsbad, CA, USA). The expression of target genes was normalized to GAPDH or U6 using the 2^−ΔΔCt^ method [27] with GAPDHA and U6 as the internal references. The primers used in this study were HOTTIP forward 5′-TCTGGTATTGCCTGGAACGCCAA-3′ and reverse 5′-CAGTGGTAATCAAGTGGAGAATC-3′, miR-92a-2 forward 5′-GAGCTAGCGAATGGCACCCT-3′ and reverse 5′-GCAGGAACGAAGTCGACTTA-3′, c-Met forward 5′-GAGCCACTTAGAATCGAGGA-3′ and reverse 5′-CTGAGGCTATAGATTCGTGCC-3′, GAPDH forward 5′-CCAAGGTCATCCATGACAAC-3′ and reverse 5′-GCTTCACCACCTTCTTGATG-3′, and U6 forward 5′-AGAGAAGATTAGCATGGCCCCTG-3′ and reverse 5′-AGTGCAGGGTCCGAGGTATT-3′.

### Western Blot

Total proteins from cultured cells were extracted using RIPA Lysis Buffer (Beyotime, Shanghai, China). Approximately equal amounts of proteins were separated by 10% SDS-PAGE and transferred onto PVDF membranes. After blocking with 5% non‐fat milk, the membranes were incubated with primary antibodies against c-Met (1:1000, Abcam), Bax (1:1000, Abcam), Bcl-2 (1:1000, Abcam) and GAPDH (1:10,000, Cell Signaling Technology) overnight at 4 °C. The membranes were subsequently exposed to horseradish peroxidase (HRP)-conjugated secondary antibodies for 2 h at room temperature. The protein bands of interest were detected using the ECL kit (Millipore), and protein levels were quantified using ImageJ software.

### Luciferase Reporter Assay

Targetscan v7.2 and Starbase v.8 web tools were used to predict the binding site between HOTTIP and miR-92a-2 and between miR-92a-2 and c-Met. The wild type (WT) or mutant type (MUT) 3′-UTR of HOTTIP and c-Met containing the putative miR-92a-2 binding site was inserted into the pmirGLO vectors (Promega, Madison, WI, USA). Then mouse cardiomyocytes were co-transfected with luciferase reporter vectors and miR-92a-2 or miR-NC using Lipofectamine 2000 (Invitrogen). At 48 h post-transfection, relative luciferase activity was evaluated by the dual-luciferase reporter system.

### Apoptosis Assay

Cardiomyocyte apoptosis was evaluated with Annexin V-FITC Apoptosis Kit (BD Biosciences). Briefly, cardiomyocytes under normoxia or hypoxia conditions were stained with Annexin V-fluorescein isothiocyanate (FITC) and propidium iodide (PI) and then analyzed by flow cytometry without re-oxygenation.

### Echocardiographic Evaluation

The day before euthanization, mice in different groups were subjected to M-mode echocardiography to analyze left ventricular ejection fraction (LVEF), left ventricular fractional shortening (LVFS), left ventricular end-systolic diameter (LVESd) and left ventricular end-diastolic diameter (LVEDd) with ATL HDI 5000 ultrasound system with parameters of 15 MHz linear and 12-MHz sectorial scan heads.

### Quantification of Infarct Size

After euthanization, the myocardial tissues of mice were collected, cut into 5-μm-thick sections, embedded with paraffin, and stained with TTC solution for 15 min. After fixation with formalin, samples were photographed using a digital camera, and infarct size was analyzed using Image J software (Olympus).

### Immunohistochemistry (IHC) and Immunofluorescence (IF) Staining

IHC and IF staining were performed as previously described [28]. Briefly, 5-µm-thick paraffin-embedded sections of myocardial tissues were incubated with primary antibodies against c-Met (1:200, Abcam) and Cas-3 (1:200, Abcam) overnight at 4 °C respectively. After washed with PBS, the protein signals were visualized by incubating with Alexa FluorVR 647-labeled goat anti-rabbit IgG (ab150083, 1:1500, Abcam) and biotin-labeled goat anti-mouse lgG (ZSGB-Bio, Beijing, China) as secondary antibodies for 60 min. The samples were covered using anti-fluorescence quenching tablets containing 4,6-diamidino-2-phenylindole (DAPI). The immunohistochemistry staining was stopped with a DAB chromogen kit (ZSGB-BIO, Beijing, China) before hematoxylin staining. The positive staining signals were observed under a fluorescence microscope (Nikon, Japan).

### TUNEL Assay and H&E Staining

Some 5 µm-thick paraffin-embedded sections of myocardial tissues were incubated with recombinant terminal deoxynucleotidyl transferase (rTdT) solution at 37 °C for 1 h in the dark and stained with DAPI solution. TUNEL stained cells were observed under a fluorescence microscope. The apoptotic index was evaluated as the percentage of TUNEL-positive cells to the total cells. In addition, some sections were stained with hematoxylin–eosin solution and observed under an optical microscope as previously described [29].

### Detection of CK-MB and LDH in Serum

Serum creatine kinase MB (CK-MB) and lactate dehydrogenase (LDH) levels in mice from different groups were detected using a Creatine Kinase Activity Assay kit and a LDH Colorimetric Assay kit, respectively.

### Statistical Analysis

Data were presented as mean ± SD derived from at least three independent experiments and statistically analyzed using GraphPad Prism software. Differences were determined by Student’s *t* test (two groups) or one-way ANOVA (among three or more groups). *P* < 0.05 was considered statistically significant.

## Results

### LncRNA HOTTIP is Significantly Upregulated in the Clinical Serum Samples, Myocardial Tissues of MI Mice, and Hypoxia-Treated Cardiomyocytes

**Fig. 1:**
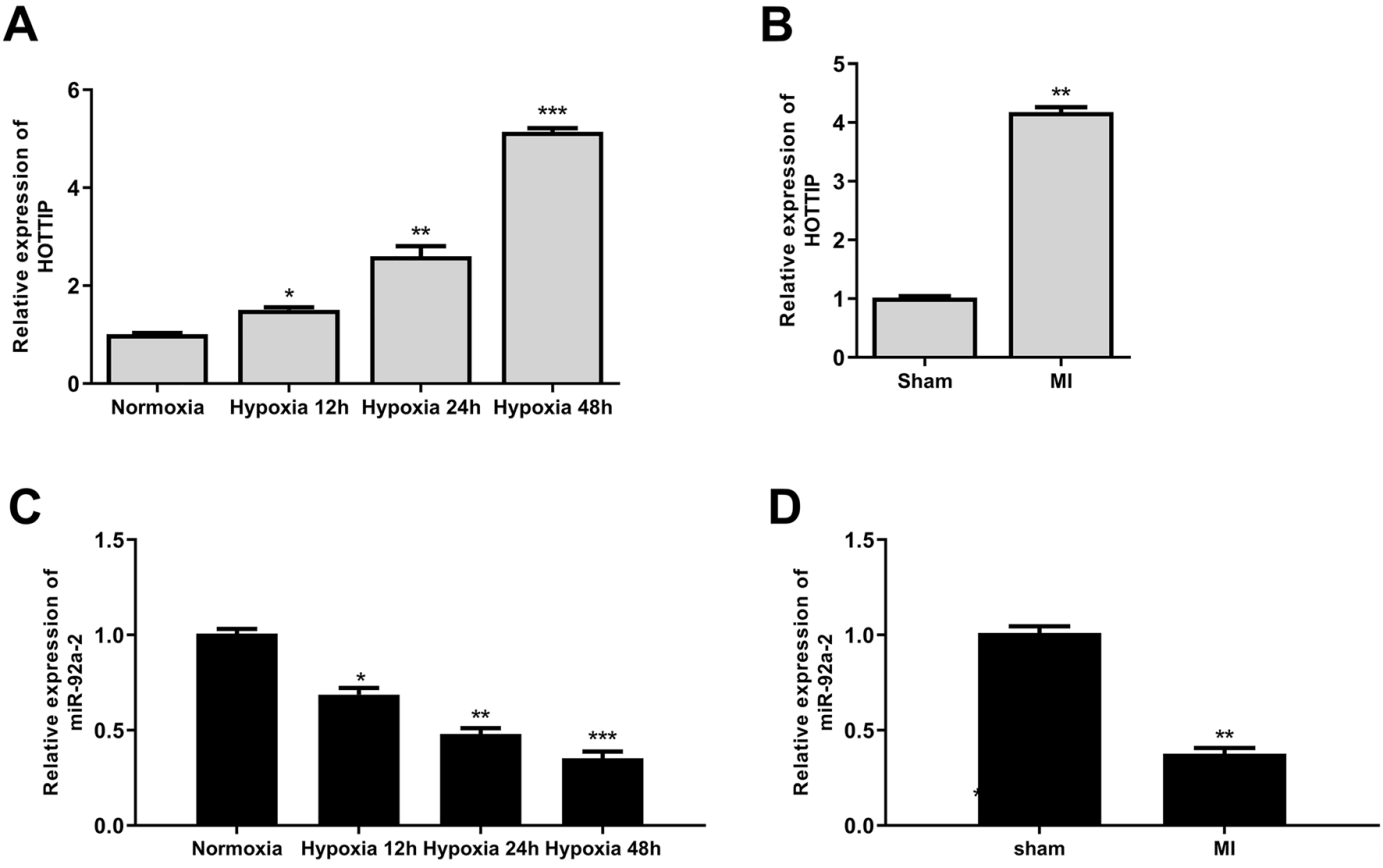
Ischemic cardiac injury increased HOTTIP expression and decreased miR-92a-2 expression. **A** and **C** HOTTIP **A** and miR-92a-2 **C** mRNA levels in hypoxia-treated mouse cardiomyocytes were evaluated by qRT-PCR (n = 3). **B** and **D** HOTTIP **B** and miR-92a-2 **D** mRNA levels in myocardial tissues of MI mice were evaluated by qRT-PCR (*n* = 6). ^*^ *P* < 0.05, ^**^ *P* < 0.01, ^***^ *P* < 0.001

HOTTIP expression in the serum samples of MI patients was detected. The results showed that HOTTIP expression was upregulated in MI patients compared with the healthy controls (Fig. s1). To investigate the potential role of HOTTIP in MI, HOTTIP expression in hypoxia-treated cardiomyocytes and myocardial tissues of MI mice was evaluated. The results indicated that HOTTIP was significantly upregulated in hypoxia-treated cardiomyocytes in a time-dependent manner (*p* < 0.05) (Fig. 1A) and in myocardial tissues of MI mice compared with the sham mice (*p* < 0.01) (Fig. 1B). In addition, miR-92a-2 was downregulated in mouse cardiomyocytes exposed to hypoxia in a time-dependent manner (*p* < 0.05) (Fig. 1C). Consistent with the findings in vitro, miR-92a-2 was also obviously downregulated in myocardial tissues of MI mice compared with the sham mice (*p* < 0.01) (Fig. 1D). These results suggested that HOTTIP and miR-92a-2 might play crucial roles in ischemic cardiac injury.

### HOTTIP Serves as a Sponge of miR-92a-2

**Fig. 2:**
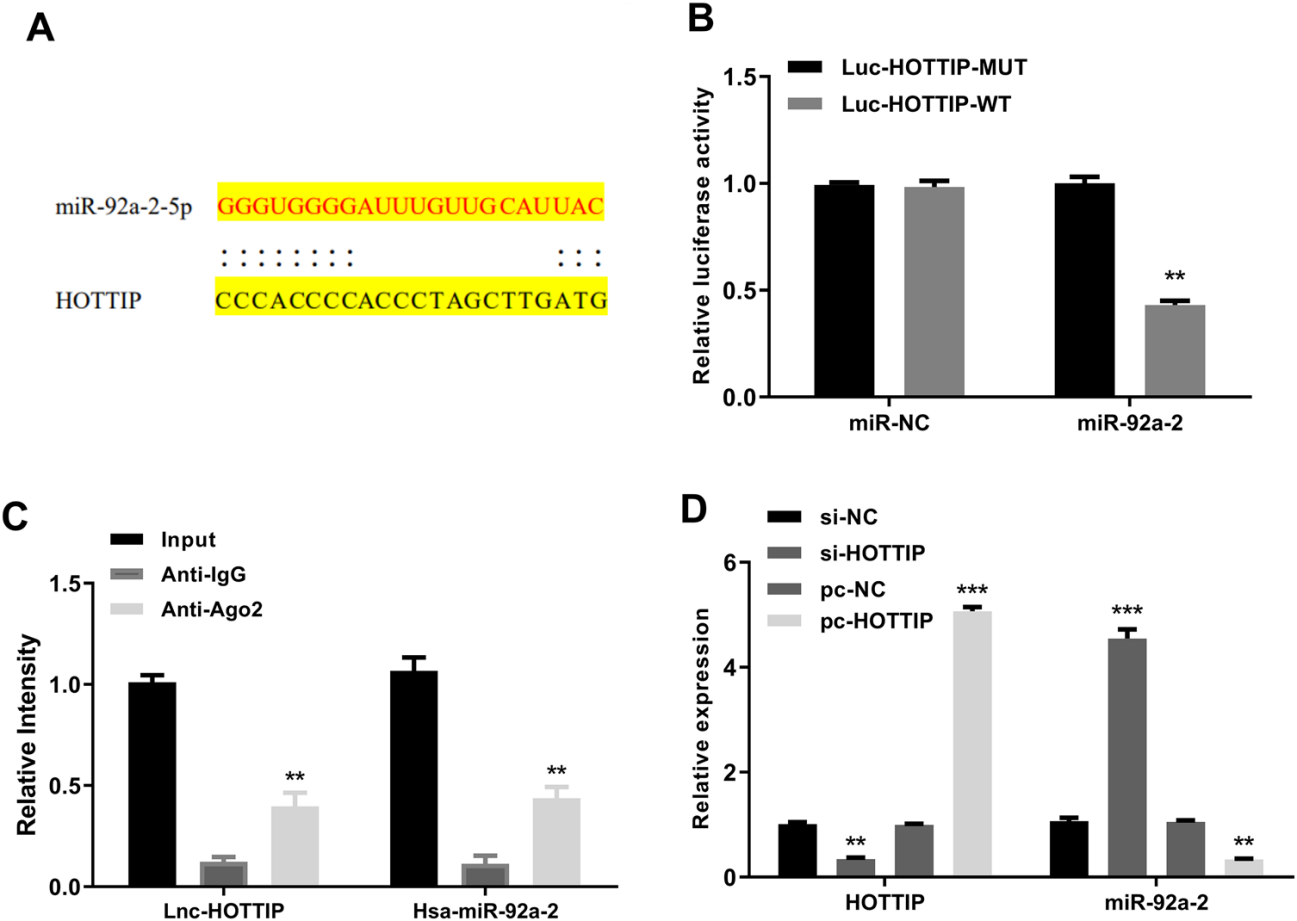
LncRNA HOTTIP serves as a sponge of miR-92a-2. **A** The putative interaction region between HOTTIP and miR-92a-2 was predicted by Starbase. **B** WT and MUT 3'UTR of HOTTIP were co-transfected with miR-92a-2 mimics or miR-NC into cardiomyocytes, and relative luciferase activity was evaluated by dual-luciferase reporter system. **C** The relative enrichment of HOTTIP and miR-92a-2 was determined using anti-Ago2 RIP assay, with anti-IgG as the negative control. **D** Cardiomyocytes were transfected with si-HOTTIP, si-NC, pc-HOTTIP, or pc-NC, and the mRNA expression of HOTTIP and miR-92a-2 was evaluated by qRT-PCR. *n* = 3, ^**^ *P* < 0.01, ^***^ *P* < 0.001

The potential targets of HOTTIP were predicted using Starbase. The results revealed a putative binding site between HOTTIP and miR-92a-2 (Fig. 2A), suggesting that miR-92a-2 might be a target of HOTTIP. Luciferase reporter assay was performed and indicated that miR-92a-2 mimics significantly decreased the relative luciferase activity of WT HOTTIP vector (*p* < 0.01), while exhibited no impact on the MUT vector (Fig. 2B). Meanwhile, RIP assay using anti-Ago2 was carried out and found that both HOTTIP and miR-92a-2 were significantly enriched in immune-precipitates containing anti-Ago2 compared with that containing negative control anti-IgG (*p* < 0.01) (Fig. 2C). In addition, mouse cardiomyocytes were transfected with si-HOTTIP, si-NC, pc-HOTTIP or pc-NC, and the expression of HOTTIP and miR-92a-2 was evaluated by qRT-PCR. The results showed that si-HOTTIP significantly decreased HOTTIP expression (*p* < 0.01) and increased miR-92a-2 expression compared with si-NC (*p* < 0.001), while pc-HOTTIP markedly increased HOTTIP expression (*p* < 0.001) and decreased miR-92a-2 expression compared with pc-NC (*p* < 0.01) (Fig. 2D). Furthermore, the expression level of MiR-17-92a-1 Cluster Host Gene (MIR17HG) did not decrease in the myocardial tissues of MI mice (Fig. s2). These results suggested that HOTTIP plays its role in MI via directly sponging miR-92a-2 to decrease miR-92a-2 level.

### C-Met is a Target of miR-92a-2

**Fig. 3:**
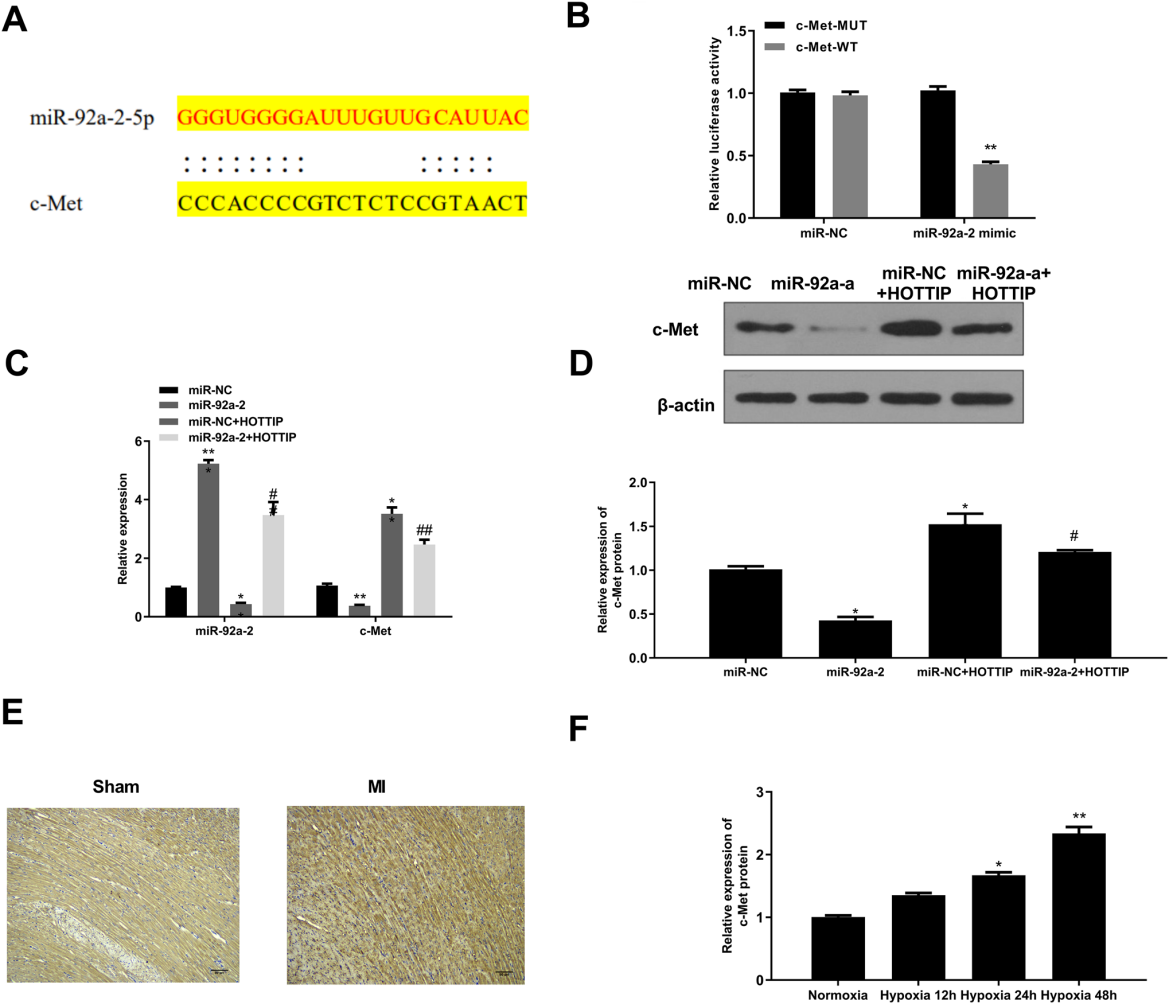
C-Met is a target of miR-92a-2. **A** The putative binding region between miR-92a-2 and c-Met was predicted by Targetscan. **B** Relative luciferase activity was evaluated by dual-luciferase reporter system (*n* = 3). **C** and **D** Mouse cardiomyocytes were transfected with miR-92a-2 mimics, miR-NC, or co-transfected with miR-NC and pc-HOTTIP, or miR-92a-2 mimics and pc-HOTTIP. **C** MiR-92a-2 and c-Met levels were evaluated by qRT-PCR (*n* = 3). **D** c-Met protein level was evaluated by Western blot (*n* = 3). **E** c-Met expression in myocardial tissues of MI and sham mice was observed by IHC assay (*n* = 6). **F** c-Met mRNA level in hypoxia-treated mouse cardiomyocytes was evaluated by qRT-PCR (*n* = 3). ^*^ *P* < 0.05, ^**^ *P* < 0.01, ^***^ *P* < 0.001 *vs.* miR-NC or normoxia condition. ^#^ *P* < 0.05, ^##^ *P* < 0.01 *vs.* miR-92a-2 mimics group

Next, Targetscan was applied to predict the potential targets of mIR-92a-2, and the results indicated that c-Met might be a target of miR-92a-2 (Fig. 3A). Luciferase reporter assay showed that miR-92a-2 mimics significantly decreased the relative luciferase activity of WT c-Met vector compared with miR-NC (*p* < 0.01) but had no obvious effect on that of MUT c-Met vector (Fig. 3B). Mouse cardiomyocytes were transfected with miR-92a-2 mimics, miR-NC, or co-transfected with miR-NC and pc-HOTTIP or miR-92a-2 mimics and pc-HOTTIP. QRT-PCR indicated that miR-92a-2 mimics significantly increased miR-92a-2 expression compared with miR-NC (*p* < 0.001), and pc-HOTTIP decreased miR-92a-2 expression compared with miR-NC (*p* < 0.01), while co-transfection of miR-92a-2 mimics and pc-HOTTIP obviously reversed the effect of miR-92a-2 mimics (*p* < 0.01) (Fig. 3C). Meanwhile, miR-92a-2 mimics markedly decreased c-Met expression at both mRNA (*p* < 0.01) (Fig. 3C) and protein (*p* < 0.01) (Fig. 3D) levels compared with miR-NC. HOTTIP overexpression significantly increased c-Met expression at both mRNA (*p* < 0.01) (Fig. 3C) and protein (*p* < 0.01) (Fig. 3D) levels compared with miR-NC. By contrast, co-transfection of miR-92a-2 mimics and pc-HOTTIP obviously reversed the effect of miR-92a-2 mimics on c-Met expression at mRNA level (*p* < 0.01) (Fig. 3C) and protein level (*p* < 0.05) (Fig. 3D). In addition, IHC assay showed that c-Met expression was obviously increased in myocardial tissues of MI mice compared with that of the sham mice (Fig. 3E). Similarly, c-Met expression was increased in hypoxia-treated cardiomyocytes in a time-dependent manner (*p* < 0.05) (Fig. 3F). These results suggested that HOTTIP might elevate c-Met expression through sponging miR-92a-2 in MI.

### HOTTIP Knockdown Promotes Growth and Inhibits Apoptosis of Hypoxia-Treated Cardiomyocytes In Vitro

**Fig. 4:**
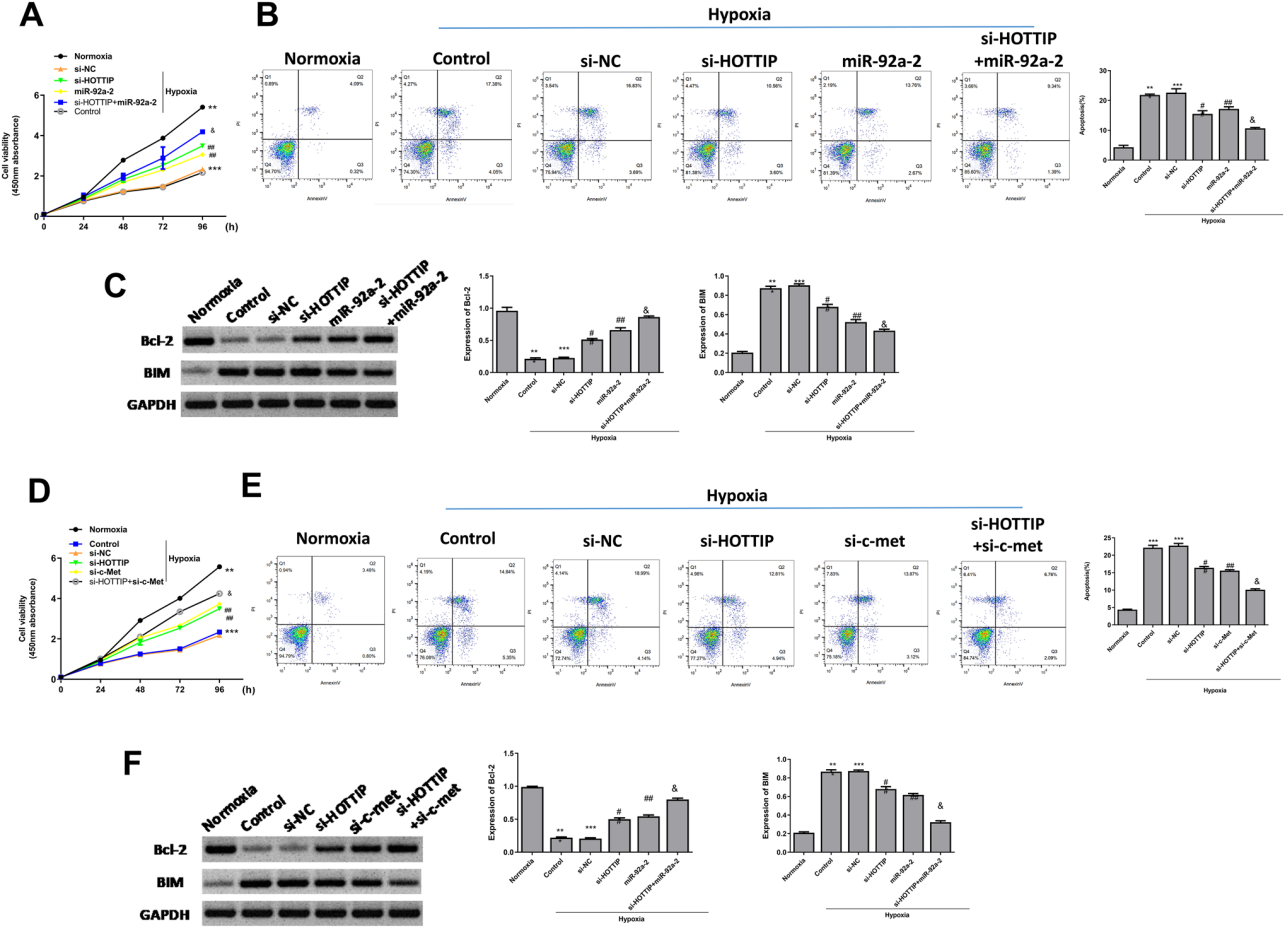
HOTTIP knockdown promotes growth and inhibits apoptosis of hypoxia-treated cardiomyocytes in vitro. **A** and **B** Mouse cardiomyocytes were transfected with si-HOTTIP, si-NC, miR-92a-2 mimics, or co-transfected with miR-92a-2 mimics and si-HOTTIP and then treated with hypoxia or normoxia conditions. **A** Cell viability was evaluated by CCK-8 assay. **B** Cell apoptosis was evaluated by flow cytometry assay. **C** Expressions of bcl-2 and BIM were evaluated by Western blot. **D** and **E** Mouse cardiomyocytes were transfected with si-HOTTIP, si-NC, si–c-Met, or co-transfected with si-HOTTIP and si–c-Met, and then treated with hypoxia or normoxia conditions. **D** Cell viability was evaluated by CCK-8 assay. **E** Cell apoptosis was evaluated by flow cytometry assay. **F** Expressions of bcl-2 and BIM were evaluated by Western blot. *n* = 3, ^**^ *P* < 0.01, ^***^ *P* < 0.001 *vs.* normoxia condition; ^##^ *P* < 0.01 *vs.* si-NC group; & *P* < 0.05 *vs.* si-HOTTIP group

Next, cardiomyocytes were transfected with si-HOTTIP, si-NC, miR-92a-2 mimics, or co-transfected with miR-92a-2 mimics and si-HOTTIP, and cell viability and apoptosis were evaluated. As shown in Fig. 4A and Fig. 4B, hypoxia significantly decreased viability and induced apoptosis of cardiomyocytes compared with normoxia condition (*p* < 0.001), and si-HOTTIP and miR-92a-2 mimics significantly promoted viability and inhibited apoptosis of hypoxia-treated cardiomyocytes compared with si-NC (*p* < 0.01), and co-transfection of miR-92a-2 mimics and si-HOTTIP further enhanced cell viability and inhibited apoptosis of hypoxia-treated cardiomyocytes compared with si-HOTTIP group (*p* < 0.05). We further detected the expression of Bcl-2 and BIM. The results showed that hypoxia significantly decreased bcl-2 expression and induced BIM expression compared with normoxia condition (*p* < 0.001), and si-HOTTIP and miR-92a-2 mimics significantly promoted bcl-2 expression and inhibited BIM expression compared with si-NC in hypoxia-treated cardiomyocytes (*p* < 0.01). Furthermore, co-transfection of miR-92a-2 mimics and si-HOTTIP further enhanced the effect of si-HOTTIP (Fig. 4C, *p* < 0.05). Similarly, the results of CCK-8 assay (Fig. 4D) and flow cytometry assay (Fig. 4E) showed that both si–c-Met and si-HOTTIP significantly promoted viability and inhibited apoptosis of hypoxia-treated cardiomyocytes compared with si-NC (*p* < 0.01), and co-transfection of si–c-Met and si-HOTTIP further enhanced cell viability and inhibited apoptosis of hypoxia-treated cardiomyocytes compared with si-HOTTIP group (*p* < 0.05). We also found that both si–c-Met and si-HOTTIP significantly promoted bcl-2 expression and inhibited BIM expression in hypoxia-treated cardiomyocytes compared with si-NC (Fig. 4F, *p* < 0.01). We further demonstrated that c-Met overexpression reversed the protective effect of si-HOTTIP or miR-92a-2 mimics on AMI progression, including cardiomyocyte apoptosis (Fig. s3). These results indicated that HOTTIP knockdown promoted growth and inhibited apoptosis of hypoxia-treated cardiomyocytes by modulating the miR-92a-2/c-Met axis in vitro.

### HOTTIP Knockdown Alleviates Myocardial Function Through Upregulating miR-92a-2 In Vivo

**Fig. 5:**
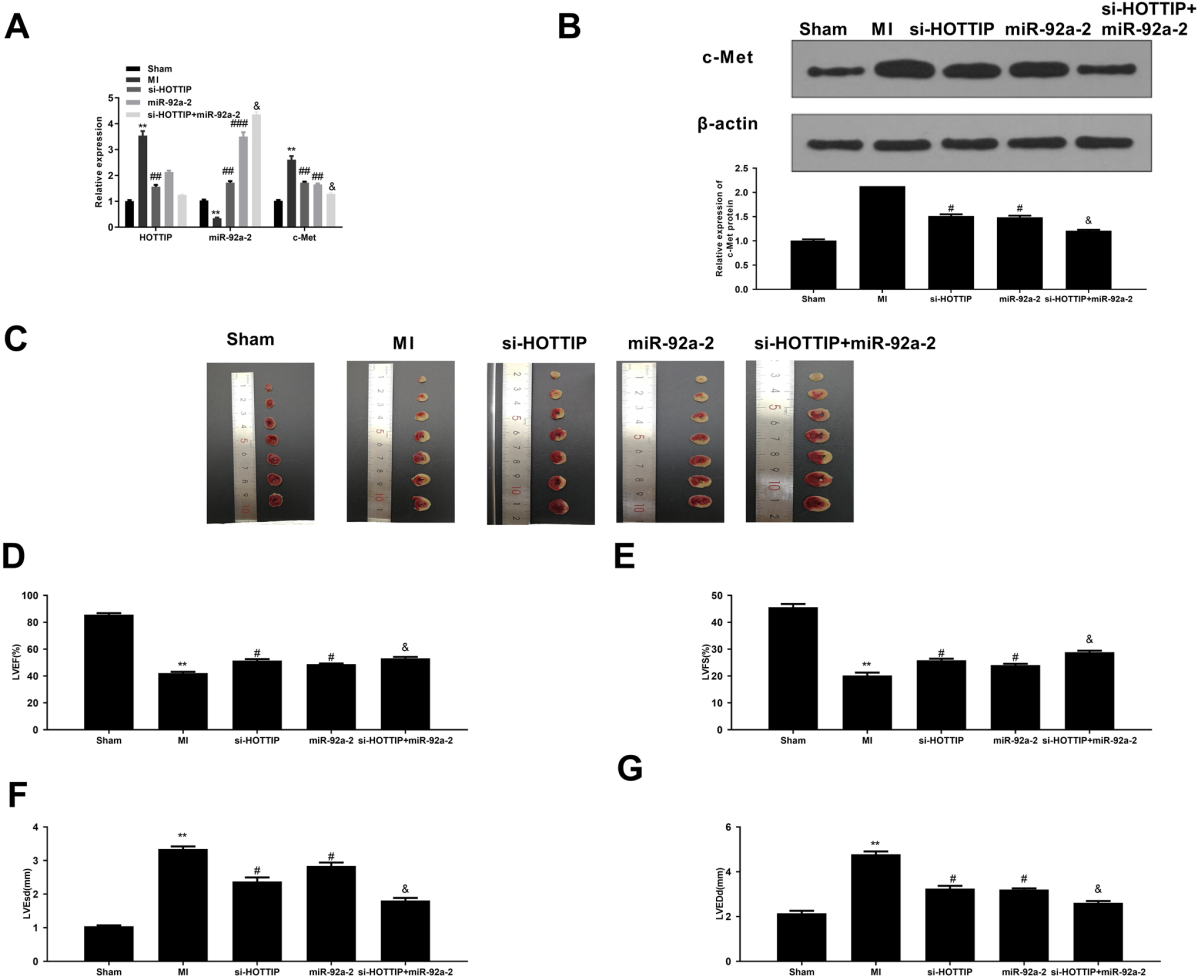
HOTTIP knockdown alleviates myocardial function via upregulating miR-92a-2 in vivo. **A** The mRNA levels of HOTTIP, miR-92a-2 and c-Met in myocardial tissues of mice from different groups were evaluated by qRT-PCR. **B** c-Met protein levels in myocardial tissues of mice from different groups were evaluated by Western blot. **C** The infarct sizes of mice from different groups were evaluated by TTC staining assay. **D**–**G** Cardiac injury indexes including LVEF **D**, LVFS **E**, LVESd **F**, and LVEDd **G** of different groups were evaluated by echocardiographic measurement. ^**^ *P* < 0.01 *vs.* sham group; ^#^ *P* < 0.05 *vs.* MI group; ^&^ *P* < 0.05 *vs.* si-HOTTIP group

To further determine the cardio-protective effects of HOTTIP in vivo, si-HOTTIP, miR-92a-2 mimics, or si-HOTTIP plus miR-92a-2 mimics were injected into mice through intramyocardial injection. The results of qRT-PCR showed that si-HOTTOP significantly decreased HOTTIP expression (*p* < 0.01), increased miR-92a-2 expression (*p* < 0.01), and decreased c-Met expression (*p* < 0.01); MiR-92a-2 mimics markedly increased miR-92a-2 expression (*p* < 0.01) and decreased c-Met expression (*p* < 0.01); Co-injection of si-HOTTIP and miR-92a-2 mimics obviously further elevated the effect of si-HOTTIP on the expression of miR-92a-2 (*p* < 0.05) and c-Met (*p* < 0.05) (Fig. 5A). Meanwhile, si-HOTTIP and miR-92a-2 mimics all significantly decreased c-Met protein level (*p* < 0.05), and si-HOTTIP plus miR-92a-2 mimics further decreased c-Met protein level compared with si-HOTTIP group (*p* < 0.05) (Fig. 5B). In addition, we found that the infarct size of myocardial tissues was significantly increased in the MI group and HOTTIP knockdown and miR-92a-2 mimics obviously decreased infarct size. Meanwhile, the inhibitory effect of si-HOTTIP was significantly enhanced by miR-92a-2 mimics (Fig. 5C). In addition, cardiac function indexes including LVEF (Fig. 5D), LVFS (Fig. 5E), LVESd (Fig. 5F), and LVEDd (Fig. 5G) of different groups were evaluated by echocardiographic measurement. The results indicated that LVEF and LVFS of myocardial tissues in MI group were significantly decreased, while LVESd and LVEDd of myocardial tissues in MI group were increased compared with the sham group (*p* < 0.01). HOTTIP knockdown led to a significant increase in LVEF and LVFS and a marked decrease in LVESd and LVEDd (*p* < 0.05). Meanwhile, the protective effect of si-HOTTIP was significantly enhanced by miR-92a-2 overexpression (*p* < 0.05). These results revealed that HOTTIP downregulation could effectively alleviate myocardial dysfunction through upregulating miR-92a-2 in vivo.

### HOTTIP Knockdown Improves Myocardial Injury and Apoptosis via Sponging miR-92a-2 In Vivo

**Fig. 6:**
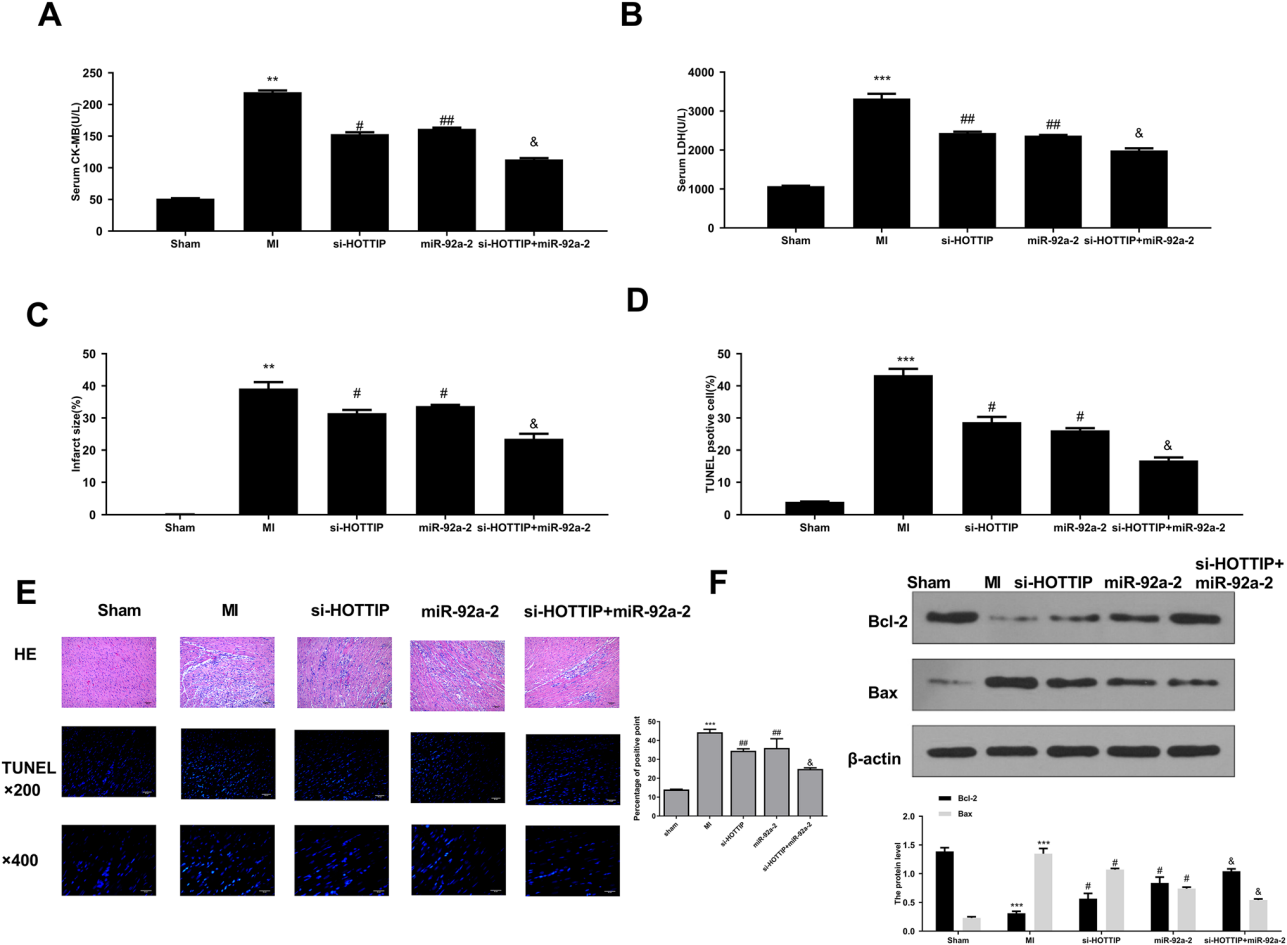
HOTTIP knockdown improves myocardial injury via sponging miR-92a-2. **A** and **B** The serum CK-MB **A** and LDH **B** levels were evaluated by respective detection kits. **C** Myocardial infarct size. **D** Apoptosis rate of cardiomyocytes was quantified from TUNEL staining assay. **E** Cardiomyocyte apoptosis was observed by TUNEL and H&E staining assay. **F** Bax and Bcl-2 protein levels in mouse heart tissues were evaluated by Western blot. *n* = 6, ^**^ *P* < 0.01, ^***^ *P* < 0.001 *vs.* sham group; ^#^ *P* < 0.05, ^##^ *P* < 0.01 *vs.* MI group; ^&^ *P* < 0.05 *vs.* si-HOTTIP group

We also evaluated the degree of myocardial cell injury by measuring serum CK-MB and LDH levels. The results (Fig. 6A and B) showed that serum CK-MB and LDH levels were significantly increased in the MI group than in the sham group (*p* < 0.001), and both si-HOTTIP and miR-92a-2 mimics markedly decreased serum CK-MB and LDH levels (*p* < 0.01). Meanwhile, miR-92a-2 mimics significantly enhanced the inhibitory effect of si-HOTTIP on serum CK-MB and LDH levels (*p* < 0.05). The myocardial infarct size was increased in all groups except for the sham group (*p* < 0.05). Si-HOTTIP and miR-92a-2 mimics significantly decreased the myocardial infarct size (*p* < 0.05), while the inhibitory effect of si-HOTTIP was enhanced by miR-92a-2 mimics (*p* < 0.05) (Fig. 6C). TUNEL staining assay was used to evaluate cardiomyocyte apoptosis rate in mouse heart tissues, and the results indicated that apoptosis rate was significantly increased in MI group than in the sham group (*p* < 0.05). Both si-HOTTIP and miR-92a-2 mimics markedly decreased apoptosis in MI mice (*p* < 0.05). Meanwhile, the inhibitory effect of si-HOTTIP was obviously enhanced by miR-92a-2 mimics (*p* < 0.05) (Fig. 6D and E). H&E staining assay (Fig. 6E) also showed a similar trend with TUNEL staining assay. In other words, cardiomyocyte apoptosis was significantly increased in MI group than in the sham group, and both si-HOTTIP and miR-92a-2 mimics obviously inhibited cardiomyocyte apoptosis. Furthermore, the protective effect of si-HOTTIP was obviously enhanced by miR-92a-2 mimics. IF staining also showed that Cas-3 expression in MI was inhibited when treated with si-HOTTIP or miR-92a-2 mimics (Fig. s4). In addition, the apoptosis-related makers, including pro-apoptotic factor Bax and anti-apoptotic factor Bcl-2, in mouse heart tissues was evaluated by Western blot (Fig. 6F). The results indicated that Bax was significantly upregulated (*p* < 0.001), while Bcl-2 (*p* < 0.001) was downregulated in MI mice compared with the sham mice. Si-HOTTIP and miR-92a-2 mimics markedly decreased Bax expression (*p* < 0.05) and increased Bcl-2 expression (*p* < 0.05), and si-HOTTIP plus miR-92a-2 mimics further enhanced Bcl-2 expression (*p* < 0.05) and decreased Bax expression (*p* < 0.05). These results demonstrated that HOTTIP downregulation effectively improved myocardial injury and inhibited cardiomyocyte apoptosis by targeting miR-92a-2 in vivo.

## Discussion

Increasing studies have suggested that lncRNAs play important roles in gene expression involved in the development of AMI [30]. lncRNA MIAT regulates microvascular dysfunction by acting as a competing endogenous RNA (ceRNA) of miR-150-5p [31]. LncRNA GAS5 regulates apoptosis of macrophages and vascular endothelial cells in atherosclerosis[32]. Since AMI is the first manifestation of ischemic heart disease, it has drawn more and more attention [33]. Over the past decades, studies have revealed that AMI progression, especially cardiomyocyte proliferation and apoptosis, is closely related to many lncRNAs, some of which might be potential biomarkers for AMI [34]. For instance, ANRIL knockdown relieves myocardial cell apoptosis during AMI by regulating the expression of IL-33/ST2 [35]. Inhibition of lncRNA Mirt1 significantly attenuates AMI via suppressing cardiomyocyte apoptosis and reduces inflammatory cell infiltration by inhibiting the activation of NF-κB signaling pathway [36]. LncRNA Gm2691 overexpression attenuates cardiomyocyte apoptosis and inflammatory responses after MI via modulating PI3K/Akt signaling pathway [37]. LncRNA TUG1 downregulation ameliorates myocardial injury during ischemia and reperfusion by regulating HMGB1- and Rac1-mediated autophagy via directly upregulating miR-142-3p [38]. LncRNA Kcnq1ot1 renders cardiomyocyte apoptosis during AMI via upregulating Tead1 expression by directly sponging miR-466 k and miR-466i-5p [39]. LncRNA XIST upregulation effectively inhibits hypoxia-induced cardiomyocyte apoptosis by targeting miR-150-5p/Bax axis during AMI progression [40]. In addition, lncRNAs SOX2-OT [41], RMRP [42], HULC [43] and MHRT [44] are also involved in regulating hypoxia-treated cardiomyocyte apoptosis. Although lncRNA HOTTIP has been identified to play crucial roles in coronary artery diseases, its effect and specific mechanism in AMI have not been reported. In this study, we, for the first time, revealed that HOTTIP is significantly upregulated in myocardial tissues of MI mice and hypoxia-treated cardiomyocytes, suggesting a potential role of HOTTIP in AMI.

Recent reports have also revealed that many miRNAs are involved in cardiomyocyte apoptosis during AMI. For example, miR-124 regulates oxidative stress- and hypoxia-induced cardiomyocyte apoptosis by targeting Dhcr24, suggesting that miR-124/Dhcr24 axis might be a potential biomarker for AMI [45]. MiR-155 promotes AMI-induced cardiomyocyte apoptosis through targeting QKI [46]. MiR-214 is highly expressed in the sera of elderly AMI patients, and its upregulation inhibits myocardial cell apoptosis by decreasing the expression of miR-214 target genes, including PUMA, PTEN, Bax, and caspase 7 [47]. In this study, we predicted the potential targets of lncRNA HOTTIP using Starbase v2.0 and showed that miR-92a-2 is a target of HOTTIP. Luciferase reporter assay and RIP assay also confirmed that HOTTIP could directly sponge miR-92a-2. Moreover, we found that the expression level of miR-17-92a-1 cluster host gene (MIR17HG) is not changed in the myocardial tissues of MI mice, indicating that HOTTIP specifically decreases miR-92a-2 level and miR-92a-2 level is modulated during AMI process in mice. Moreover, co-transfection of miR-92a-2 mimics and si-HOTTIP further enhances viability and inhibits apoptosis of hypoxia-treated cardiomyocytes than transfection with si-HOTTIP alone both in vitro and in vivo, suggesting the important roles of miR-92a-2 in AMI.

C-Met has been identified as a direct target of miRNAs to participate in important biological processes. Cheng et al. revealed that miR-449a inhibits hepatocellular carcinoma cell growth via G1 phase arrest and activates HGF/MET c-Met signaling pathway [48]. C-Met is involved in miR-433-mediated inhibition of the EMT process in bladder cancer through modulating the Akt/GSK-3β/Snail signaling pathway [49]. In this study, we identified c-Met as a target of miR-92a-2 and showed that miR-92a-2 mimics significantly decreases the relative luciferase activity of WT c-Met vector but not MUT c-Met vector compared with miR-NC. In addition, c-Met the expression is markedly decreased by miR-92a-2 mimics but increased by HOTTIP overexpression, and co-transfection of miR-92a-2 mimics and pc-HOTTIP obviously reverses the effect of miR-92a-2 mimics on c-Met expression. Moreover, miR-92a-2 mimics plus si-HOTTIP significantly enhance the protective effect of si-HOTTIP on myocardial function in vivo and cardiomyocytes apoptosis in vitro. Bcl-2 family comprises both cell death inhibiting and promoting proteins, such as anti-apoptotic factor Bcl-2 and pro-apoptotic factor Bax [50]. We found that si-HOTTIP and miR-92a-2 mimics markedly decrease Bax expression and increase Bcl-2 expression in mouse heart tissues, and si-HOTTIP plus miR-92a-2 mimics further enhance Bcl-2 expression and decrease Bax expression. These results demonstrated that the protective effects of si-HOTTIP and miR-92a-2 mimics in AMI are mediated by cardiomyocytes apoptosis. However, whether c-Met overexpression could reverse the protective effect of si-HOTTIP or miR-92a-2 mimics in AMI progression, including cardiomyocytes apoptosis, should be determined in the subsequent experiments. In addition, our results are obtained in mouse-derived samples and need to be further clarified in myocardial tissues of AMI patients.

## Conclusion

Our study revealed a new regulatory mechanism of lncRNA HOTTIP in AMI: lncRNA HOTTIP downregulation could effectively attenuate AMI progression via targeting the miR-92a-2/c-Met axis, suggesting that HOTTIP might be a potential therapeutic target for AMI.

## Supplementary Information

Below is the link to the electronic supplementary material.

Serum HOTTIP levels were upregulated in MI patients. ** *P* < 0.01 (tif 777 kb)

Ischemic cardiac injury did not affect miR-17-92a-1 cluster host gene (MIR17HG) expression (tif 803 kb)

HOTTIP knockdown promotes growth and inhibits apoptosis of hypoxia-treated cardiomyocytes in vitro. (A) Mouse cardiomyocytes were transfected with si-HOTTIP, pc-c-Met, miR-92a-2 mimics, co-transfected with si-HOTTIP and pc-c-Met, or co-transfected with miR-92a-2 mimics and pc-c-Met, and then treated with hypoxia or normoxia condition. (A) Cell apoptosis was evaluated by flow cytometry assay. (B) Bcl-2 and BIM expressions were evaluated by Western blot assay. *n* = 3, ** *P* < 0.01, *** *P* < 0.001 vs. normoxia condition; ## *P* < 0.01 vs. si-NC group; & *P* < 0.05 vs. si- HOTTIP group (tif 13740 kb)

HOTTIP knockdown improves myocardial injury through sponging miR-92a-2. The expression of Cas-3 was evaluated by IF staining. *N* = 6, ** *P* < 0.01, *** *P* < 0.001 vs. sham group; # *P* < 0.05, ## *P* < 0.01 vs. MI group; & *P* < 0.05 vs, si-HOTTIP group (tif 8941 kb)

## Data Availability

The datasets used and/or analyzed during the current study are available from the corresponding author on reasonable request.

## Declarations

### Research Involving Animals

All procedures were approved by the Ethical Committee of the First Affiliated Hospital of USTC, Division of Life Sciences and Medicine, University of Science and Technology of China.
