# Supplementary figures and images for: LncRNA HOTTIP Knockdown Attenuates Acute Myocardial Infarction via Regulating miR-92a-2/c-Met Axis
Source: Cardiovasc Toxicol. 2022 Jan 19;22(4):352–64. doi: 10.1007/s12012-021-09717-3 (PMC8907089; doi:10.1007/s12012-021-09717-3)

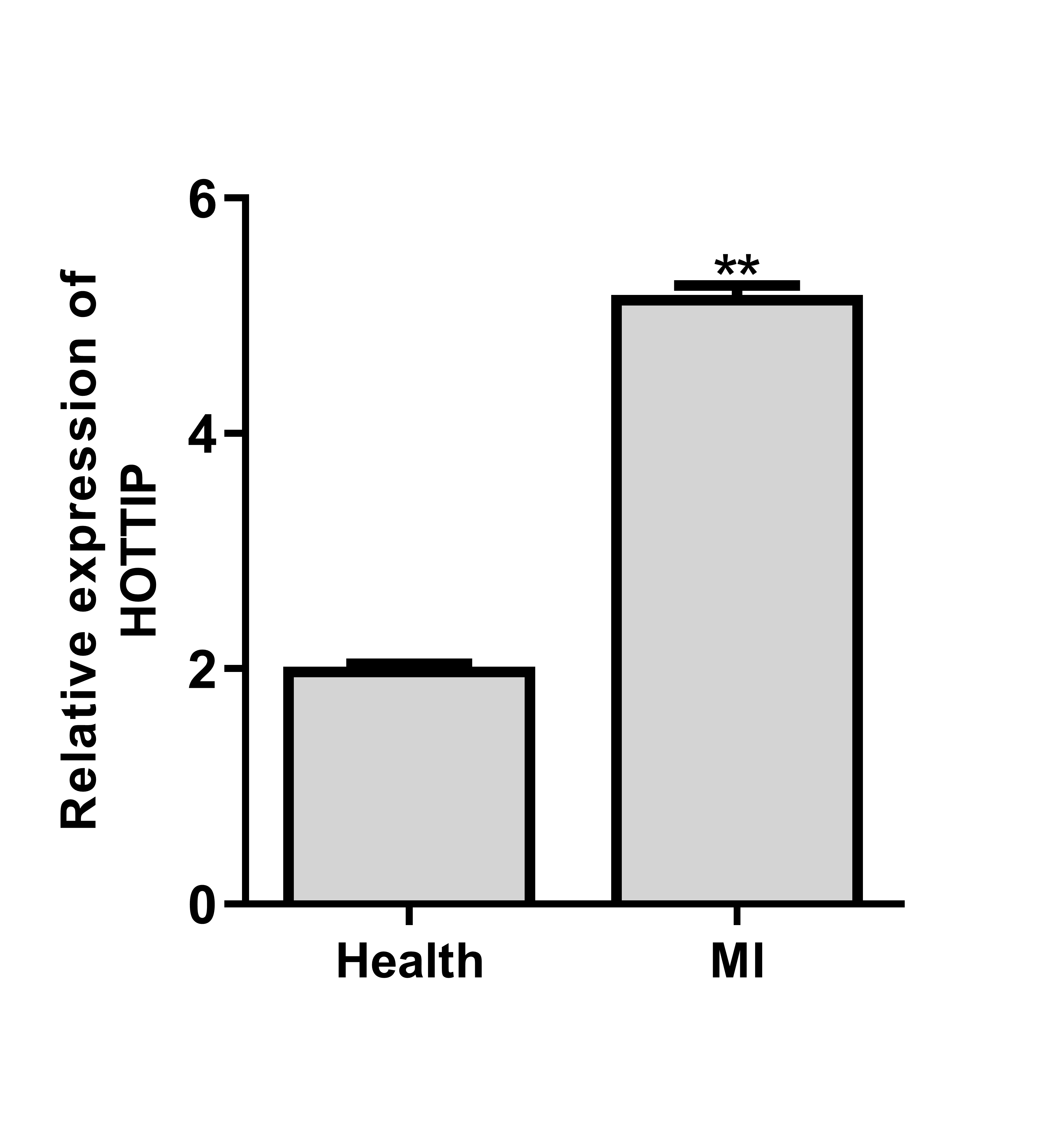

Supplement: Supplementary file 1 — Serum HOTTIP levels were upregulated in MI patients. ** P < 0.01 (tif 777 kb) [file 12012_2021_9717_MOESM1_ESM.tif]

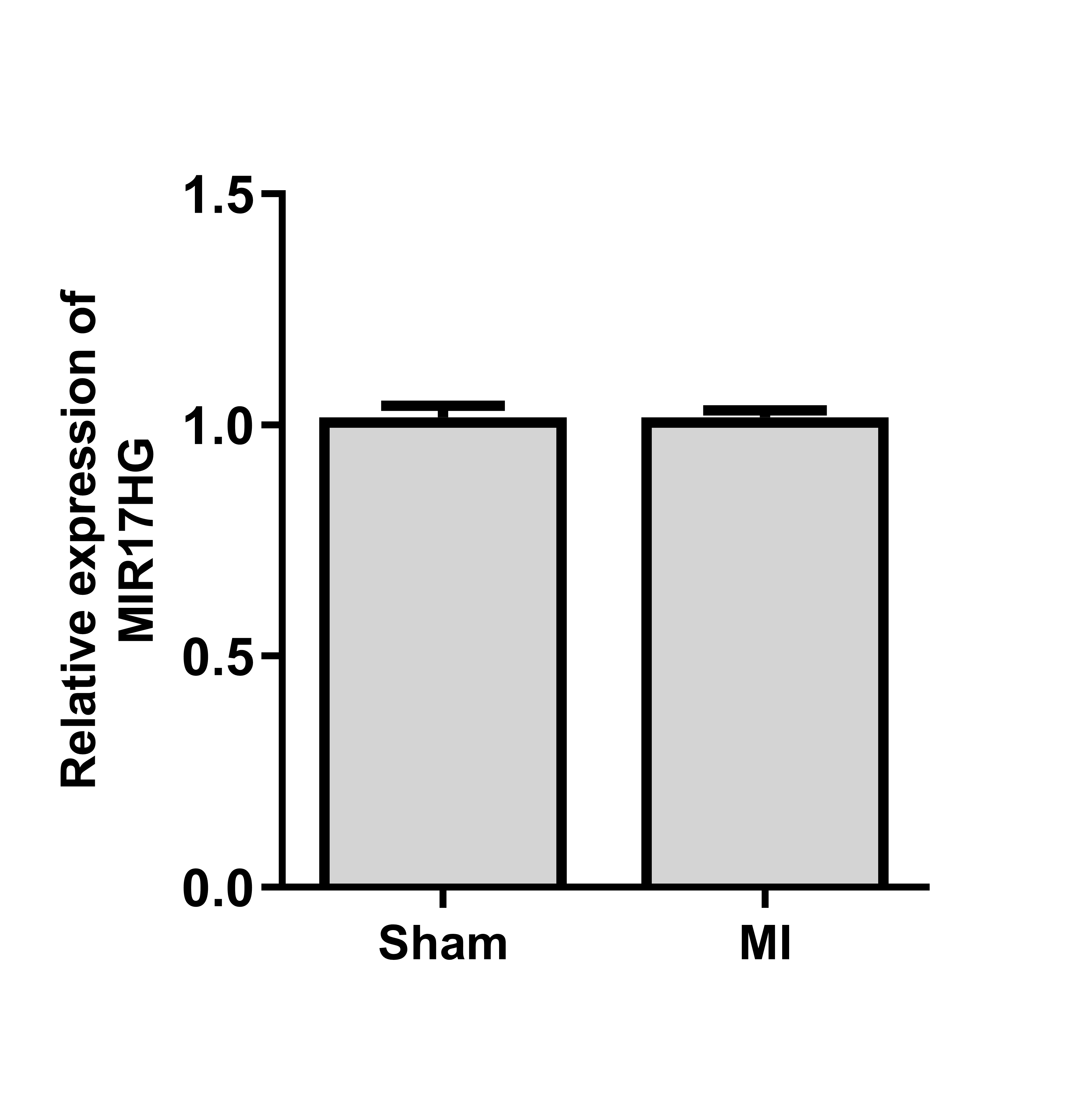

Supplement: Supplementary file 2 — Ischemic cardiac injury did not affect miR-17-92a-1 cluster host gene (MIR17HG) expression (tif 803 kb) [file 12012_2021_9717_MOESM2_ESM.tif]
